# Improved community detection in weighted bipartite networks

**DOI:** 10.1098/rsos.140536

**Published:** 2016-01-20

**Authors:** Stephen J. Beckett

**Affiliations:** Biosciences, College of Life and Environmental Sciences, University of Exeter, Exeter EX4 4QE, UK

**Keywords:** modular structure, network ecology, bipartite networks, modules

## Abstract

Real-world complex networks are composed of non-random quantitative interactions. Identifying communities of nodes that tend to interact more with each other than the network as a whole is a key research focus across multiple disciplines, yet many community detection algorithms only use information about the presence or absence of interactions between nodes. Weighted modularity is a potential method for evaluating the quality of community partitions in quantitative networks. In this framework, the optimal community partition of a network can be found by searching for the partition that maximizes modularity. Attempting to find the partition that maximizes modularity is a computationally hard problem requiring the use of algorithms. QuanBiMo is an algorithm that has been proposed to maximize weighted modularity in bipartite networks. This paper introduces two new algorithms, LPAwb+ and DIRTLPAwb+, for maximizing weighted modularity in bipartite networks. LPAwb+ and DIRTLPAwb+ robustly identify partitions with high modularity scores. DIRTLPAwb+ consistently matched or outperformed QuanBiMo, while the speed of LPAwb+ makes it an attractive choice for detecting the modularity of larger networks. Searching for modules using weighted data (rather than binary data) provides a different and potentially insightful method for evaluating network partitions.

## 1. Introduction

Bipartite networks are the representation of interactions between two distinct classes of nodes, such that nodes can only interact with nodes from the other class [1]. Such networks can be used for example to represent the way in which certain actors are related to certain events in social networks [2]; to represent industrial trade networks [3]; and in ecology to represent the interactions between plant species and pollinator species [4]. Identifying structure within networks is useful in explaining their formation, function and behaviour and is an important challenge in a diverse set of disciplines. Community detection algorithms are designed to identify clusters, or modules, of nodes within a network that are more likely to interact among themselves than with the rest of the network [5]. Modularity is an evaluation of the way in which nodes are partitioned into separate subsets, forming modules. This is done by assessing the extent to which interactions in the network occur within modules rather than between modules, relative to a null model [6,7]. One (of many) community detection methods is to find the partitioning of nodes into modules that will maximize the modularity of a network. Several modularity maximization algorithms have been designed to attempt to achieve this [5].

Modularity maximization was originally developed for unipartite (in which all nodes are allowed to interact with one another) networks [6]. Modularity is highest when each module appears isolated from the rest of the network. This occurs when nodes interact often with nodes in the same module and there are few between module interactions. Negative modularity scores imply that fewer interactions occur within modules than expected in a random network. But, positive modularity indicates that within module connectivity is higher than expected. The smallest and largest possible modularity scores that can be found are network dependent [1].

There are several definitions of modularity used in bipartite networks. Guimerà's modularity [8] and Barber's modularity [7] were recently reviewed [9] in the context of ecological networks. Guimerà's modularity uses weighted projections to identify separate communities within each node type. In contrast, Barber's modularity identifies joint communities composed of both types of node. In this paper, I concentrate on the modularity definition proposed by Barber and its extension to weighted networks [10] to search for communities composed of both node types, which in the context of this study are communities of plants and their respective pollinators.

**Figure 1.**
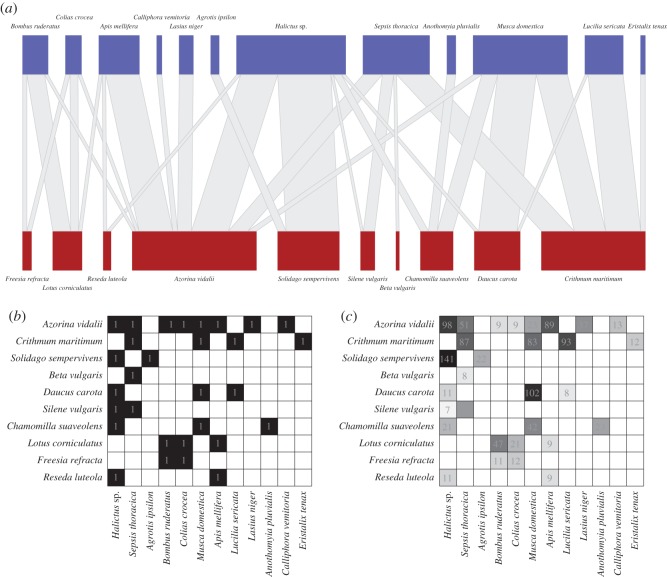
(*a*) The olesen2002flores bipartite network of 12 species of pollinators (blue nodes (top)) visiting 10 plant species (red nodes (bottom)). The width of the edges linking the nodes represents the number of pollinator–plant visitations, while the width of the nodes represents the marginal total of visits made by a pollinator species or received by a plant species.(*b*) The same network represented by the incidence matrix denoted $\tilde{A}$ in the text, where the plant species are represented as rows and the pollinator species as columns and the presence of visitations between a pollinator and plant species is represented by a 1. (*c*) The incidence matrix $\tilde{A}$ is the binary equivalent of $\tilde{W}$, the weighted interaction matrix shown here. The cell numbers correspond to the number of observed pollinator–plant visitations that occurred (where there is no number in a square there were 0 visitations).

Modularity is a major feature of plant–pollinator networks [4] and may contribute to network stability in these systems. They can be represented as bipartite networks with interactions between pollinators and plants. Pollinating species cannot pollinate other pollinating species, while plants cannot visit each other—the only allowed interactions are between different plants and pollinators (an example network is shown in figure 1).

While it is possible to use interaction weights to perform community detection [10,11], the majority of approaches only focus on whether two nodes have an association, regardless of the strength of such associations [5]. Recently, QuanBiMo [10] was introduced as the first algorithm to use quantitative interaction strengths to perform community detection via maximizing weighted modularity in bipartite networks. QuanBiMo is an algorithm to maximize modularity in bipartite networks based on hierarchical random graphs [12] and simulated annealing. Many approaches to modularity maximization have been developed for binary bipartite networks, which do not necessarily reach the same result [5,13,14]. There may be danger in relying on a single approach to maximize modularity in weighted networks, but as yet there is no other method to compare the results of QuanBiMo against. It may be possible to adapt some of the methods used for detecting communities in binary networks to deal with quantitative information, rather than having to discard this important data dimension. In this paper, I consider modifying the LPAb+ algorithm [13], that uses label propagation and multi-step agglomeration to attempt to maximize modularity in binary bipartite networks. The LPAb+ algorithm has been shown to outperform seven other available methods for binary networks [13,14] while retaining fast time complexity. These qualities make it a good candidate for extension to the case of weighted networks. Additionally, both LPAb+ and QuanBiMo operate to maximize Barber's modularity in binary networks. Thus, if LPAb+ can be modified to maximize the weighted modularity function proposed by QuanBiMo, the results can be directly compared.

The definitions of binary and weighted modularity are presented. I show how to alter the LPAb+ algorithm so it can detect weighted modularity and denote this algorithm LPAwb+. A further modification allowing a more thorough search of modularity space is also presented. I call this DIRTLPAwb+. First, the performances of LPAwb+ and DIRTLPAwb+ are assessed using an ensemble of synthetic weighted networks with a given modular structure. Then, all three algorithms for maximizing weighted modularity are compared on an empirical dataset containing 23 plant–pollinator networks. I find that QuanBiMo is highly sensitive to its input parameters, which may lead to reporting of modularity far below the optimal value in a given network. QuanBiMo reported less consistent modularity scores than either LPAwb+ or DIRTLPAwb+. These experiments show that DIRTLPAwb+ and QuanBiMo performed well on smaller networks, while the speed of LPAwb+ makes it particularly suitable for use on larger datasets. The inclusion of quantitative information in networks alters the structure of detected modules, which may have implications for how modularity is used.

## 2. Material and methods

### 2.1 Modularity

#### 2.1.1 Barber's modularity

Bipartite or two-mode networks are made of two disjoint sets of nodes such that interactions only occur between nodes of opposite types. To generalize, we say there are two node types—red and blue—and that interactions are only allowed between red and blue nodes. If there are *r* nodes of the red type and *c* nodes of the blue type, the adjacency matrix *A* is given in block diagonal form as

$$A=\left(\begin{array}{cc} 0_{r\times r} & {\tilde{A}}_{r\times c}\\ {\tilde{A}}_{c\times r}^{T} & 0_{c\times c} \end{array}\right),$$

where $\tilde{A}$ is the incidence matrix describing the connections between the different types of nodes (here T indicates the matrix transpose). This formulation allows bipartite modularity to be written as [7]

2.1 $$\begin{array}{rl} Q_{B} & =\frac{1}{m}\sum_{u=1}^{r}\sum_{v=1}^{c}({\tilde{A}}_{uv}-P_{uv})\delta (g_{u},h_{v})=\frac{1}{m}\sum_{u=1}^{r}\sum_{v=1}^{c}\left({\tilde{A}}_{uv}-\frac{k_{u}d_{v}}{m}\right)\delta (g_{u},h_{v}), \end{array}$$

where *P* is the null model matrix describing the expected probability of interactions between red and blue nodes given the degree distributions of $\tilde{A}$ [7]. This is calculated by finding: *m*, the matrix fill—the number of edges in $\tilde{A}$; *k*, that describes the node degree for red nodes (the number of blue nodes each red node interacts with); and *d*, that describes the node degree for blue nodes (the number of red nodes each blue node associates with). Red node labels are denoted by *g*, while *h* are the labels for blue nodes, and the Kronecker delta function *δ*(*g*_*u*_,*h*_*v*_) is equal to one when nodes *u* and *v* are classified as being in the same module (i.e. they have the same label value) or zero otherwise.

#### 2.1.2 Weighted bipartite modularity

Weighted bipartite modularity, *Q*_W_, can be defined as [10]

2.2 $$\begin{array}{rl} Q_{W} & =\frac{1}{M}\sum_{u=1}^{r}\sum_{v=1}^{c}({\tilde{W}}_{uv}-{\tilde{E}}_{uv})\delta (g_{u},h_{v})=\frac{1}{M}\sum_{u=1}^{r}\sum_{v=1}^{c}\left({\tilde{W}}_{uv}-\frac{y_{u}z_{v}}{M}\right)\delta (g_{u},h_{v}), \end{array}$$

where $\tilde{E}$ is the matrix of the null expectations of interaction between two nodes, *y* is the row marginal totals and *z* is the column marginal totals of $\tilde{W}$, the weighted incidence matrix. In a binary network, $\tilde{W}$ is equivalent to the binary incidence matrix $\tilde{A}$, the marginal totals will equal the node degrees (*y*=*k* and *z*=*d*) and *M*, the sum of edge weights will equal *m*, the fill. Thus equation (2.2) will reduce to equation (2.1) for a binary network. Furthermore, equation (2.2) can be reformulated into its matrical form [7,15] to allow for vectorized computation as

2.3 $$Q_{W}=\frac{1}{M}\;\mathrm{tr}(R(\tilde{W}-\tilde{E})C),$$

where for a network with *F* communities, *R* is the *F*×*r* red label matrix and *C* is the *c*×*F* blue label matrix. *R* (and *C*) are binary matrices with a single 1 in each column (row) indicating which community each red (blue) node belongs to (this information is held by the red and blue labels). These definitions of weighted bipartite modularity can now be used in the modified framework of the LPAb+ algorithm.

### 2.2 Weighted modularity maximizing algorithms

#### 2.2.1 QuanBiMo

The quantitative bipartite modularity algorithm (QuanBiMo) of Dormann & Strauss [10], based on the hierarchical random graph algorithm [12], uses a simulated annealing method to attempt to maximize weighted bipartite modularity. It is a C++ routine that is available in the R package bipartite [16] through the function computeModules. The default settings available in bipartite v. 2.05 were used (*steps*=10^6^, *tolerance*=1^−10^).

#### 2.2.2 LPAwb+

The LPAwb+ algorithm is made from two stages—a ‘bottom up’ step that maximizes modularity on a node-by-node basis using label propagation; and a ‘top down’ step that joins modules together when it results in increased network modularity. A bipartite network can have at the most F=min(r,c) communities with our chosen definition of modularity. The LPAwb+ algorithm is initialized by giving a unique label to each of the nodes in the smallest of the two sets.

*Stage 1—label propagation stage—bottom up*. Asynchronous updating of blue, then red labels on the network is performed to locally maximize modularity (equation (2.2)). For a particular red node *x*, this can be written as choosing a new label *g*_*x*_ by trying to maximize the condition

2.4 $$\begin{array}{rl} g_{x} & =\left(\sum_{v=1}^{c}\left({\tilde{W}}_{xv}-\frac{y_{x}z_{v}}{M}\right)\right)\delta (g,h_{v})=\left(\sum_{v=1}^{c}{\tilde{W}}_{xv}\delta (g,h_{v})-\sum_{v=1}^{c}\left(\frac{y_{x}z_{v}}{M}\right)\delta (g,h_{v})\right). \end{array}$$

Red nodes only use information about the blue nodes to update their labels (*g*) and similarly blue node labels (*h*) are updated only using information about the red nodes. Simplifying equation (2.4) and creating an analogue for the updating rules for blue node labels leads to the following set of conditions:

2.5 $$\begin{array}{rl} g_{x}^{\mathrm{new}} & =\underset{g}{\arg\max}\left(N_{xg}-\frac{y_{x}Z_{g}}{M}\right)\\ \text{and}\;\;h_{x}^{\mathrm{new}} & =\underset{h}{\arg\max}\left(N_{xh}-\frac{Y_{h}z_{x}}{M}\right), \end{array}\}$$

where the new label assigned to node *x* of type *g* (red) or *h* (blue) is the *g* or *h* that maximizes the condition on the right-hand side (if more than one solution exists, one is chosen at random). Here, *N*_*xg*_ is the sum of interactions from nodes connecting to *x* labelled *g*, while *Z*_*g*_ is the sum of the marginal sums of blue nodes labelled *g* and *Y*_*h*_ is the sum of the marginal sums for red nodes labelled *h*. As these ‘bottom-up’ updating rules (equation (2.5)) are mutually exclusive of one another, they are applied asynchronously such that blue labels are updated, then red nodes are updated, then blue nodes are updated and so on until modularity (equation (2.2)) can no longer be increased.

*Stage 2—agglomeration stage—top down*. When modularity can no longer be increased via stage 1's ‘bottom-up’ steps, a localized maximum of modularity for the network is reached; however this may not be the global maximum. The second stage seeks to prevent the algorithm getting stuck at local maxima by merging groups of communities together. Each identified community module *t* is composed of blue and red nodes that share the same label, i.e. when *g*_*u*_=*h*_*v*_. If there are *F* communities in total, then the merging of two different communities *t*_*i*_ and *t*_*j*_ can only occur if this would result in an increase in network modularity and if there is no third community *t*_*k*_ (1≤*k*≤*F*, *i*≠*j*≠*k*) whose merger with either of *t*_*i*_ or *t*_*j*_ would result in a larger increase to modularity. Pseudo-code representing these steps is shown in algorithm 2. Algorithm 1


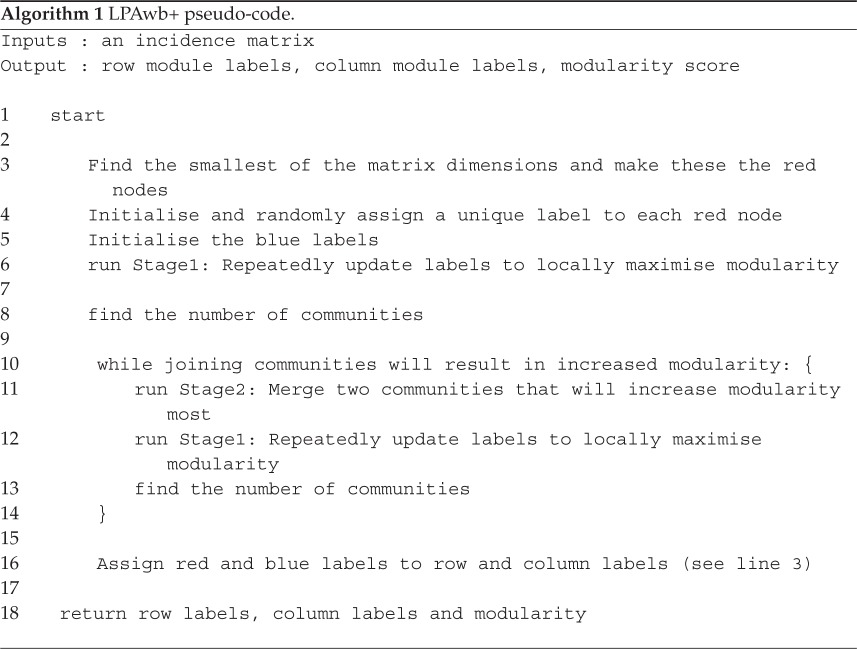


Once this merger of modules is completed, stage 1 and then stage 2 are repetitively performed until it is no longer possible to increase network modularity by merging any of the possible communities together. These modules (communities) and the modularity of this partition are the solution provided by the LPAwb+ algorithm. A key feature of the LPAwb+ algorithm is that it simplifies to the previously described LPAb+ algorithm [13] when a binary network is used as input. The LPAwb+ algorithm is stochastic—this can lead to different values of modularity being reported. To combat this issue, it has been suggested that the LPAb+ algorithm is run multiple times on a given network to find the greatest modularity score [13]. Algorithm 2


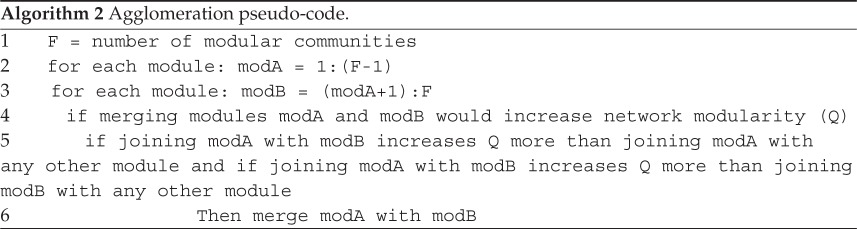


#### 2.2.3 DIRTLPAwb+

Exploratory research with QuanBiMo and LPAwb+ revealed LPAwb+ often got stuck in a suboptimal solution with a larger number of modules, when compared with QuanBiMo, as LPAwb+ starts by identifying the largest possible number of modules, then iteratively merges them until modularity cannot be increased.

Knowing that LPAwb+ is sensitive to node label initialization [13] and that it performs faster than QuanBiMo, I designed a new algorithm, DIRTLPAwb+ (see algorithm 3). DIRTLPAwb+ computes LPAwb+ multiple times with different random initializations of node labels chosen from *μ* unique possible labels; and returns the solution which finds the greatest modularity score.

DIRTLPAwb+ takes three inputs: the incidence matrix for the network of interest, the number of times that LPAwb+ should be run for each value of *μ*, and the minimum number of unique labels (modules) to start running LPAwb+ with. Therefore, *μ* ranges between this minimum value and the number of modules returned by a single execution of the LPAwb+ algorithm (when each node is initialized with a unique label) which is used as an upper limit. Algorithm 3


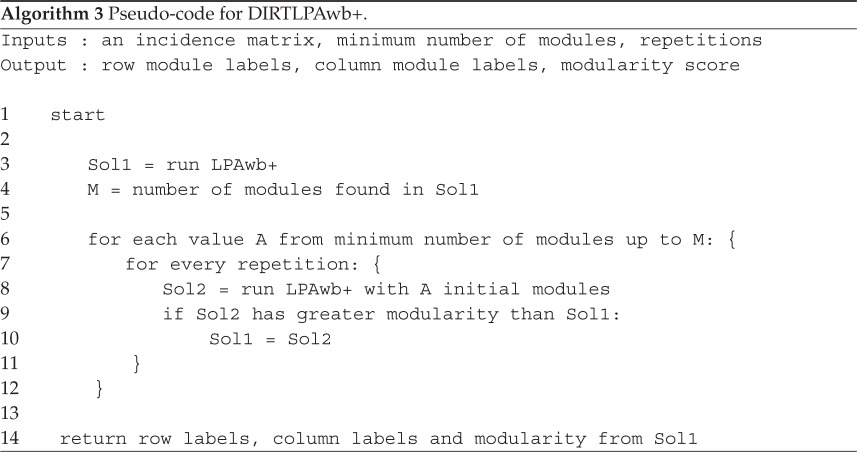


Setting the minimum number of modules to search for small, and the number of repetitions high will increase the chance of detecting the global modularity optimum for a network; but is likely to be computationally costly. I chose to give DIRTLPAwb+ default settings of 10 repetitions for each value of *μ*, starting from a minimum of four modules (note this does not preclude solutions with fewer modules being identified due to the merging process in LPAwb+) as the speed taken to perform these calculations appeared favourable to QuanBiMo for the test datasets.

### 2.3 Comparing modularity

#### 2.3.1 Normalized modularity

The modularity values of *Q*_B_ and *Q*_W_ found above are network-specific—properties such as the size and number of links in a network affect the magnitude of modularity that can be found [1,9,10]. In order to compare the strength of assortative mixing across different network studies it is necessary to account for the possibility of these effects. Dormann & Strauss [10] recommend using a null model to generate an ensemble of networks from which the standardized effect size of modularity can be assessed as a *z*-score. However, it is unclear what would make an appropriate null model for weighted networks. An alternative method is to normalize the modularity values by the maximum value that modularity can take, found in the ‘perfectly mixed’ network, in which all edges are assigned to a module and there are no links between different modules [1]. Extending this for weighted bipartite networks gives

2.6 $$Q^{\max}=\frac{1}{M}\left(M-\sum_{u=1}^{r}\sum_{v=1}^{c}\frac{y_{u}z_{v}}{M}\right)\delta (g_{u},h_{v}),$$

where as before *M* is the sum of the edges in the incidence matrix with marginal row totals *y* and marginal column totals *z*. Then normalized modularity is found as

2.7 $$Q^{\mathrm{norm}}=\frac{Q}{Q^{\max}}.$$

#### 2.3.2 Realized modularity

Realized modularity [17] has been suggested as a posterior measure of modularity that classifies the proportion of links in a network that are within, rather than between, modules. Here, I extend this measure so it can be applied to weighted as well as binary networks. If *M* is the sum of all edge weights in a network and *H* is the sum of all within-module edge weights, then realized weighted modularity is expressed as

2.8 $$Q_{R}^{\prime }=2\left(\frac{H}{M}\right)-1.$$

$Q_{R}^{\prime }$ takes values between −1, indicating that no edges exist between nodes in the same module, and 1, when all edges are interactions within modules. If *Q*′_R_=0, half of the edge weights in the network are found connecting nodes within the same module and the remaining edge weights are node connections between different modules. Note that in a weighted network $Q_{R}^{\prime }$ says nothing about the actual number of edges between or within modules, only the strength of the connecting edges.

#### 2.3.3 Normalized mutual information

The normalized mutual information (NMI) criterion is used as a way to compare the similarity of network structures found by different community detection methods [9,18]. For two different partitions *A* and *B* of the same network with a total of *n* nodes (red and blue), with *C*_*A*_ and *C*_B_ modules, respectively, the NMI is

2.9 $$\mathrm{NMI}(A;B)=\frac{-2\sum_{i=1}^{C_{A}}\sum_{j=1}^{C_{B}}N_{ij}\log(N_{ij}n/N_{i}N_{j})}{\sum_{i=1}^{C_{A}}N_{i}\log(N_{i}/n)+\sum_{j=1}^{C_{B}}N_{j}\log(N_{j}/n)},$$

where *N* is the confusion matrix with elements *N*_*ij*_ which indicate the number of nodes that appear in the *i*th module of partition *A* and the *j*th module of partition *B*; *N*_*i*_ is the number of nodes in module *i* of partition *A* and *N*_*j*_ is the number of nodes in module *j* of partition *B*. If NMI(*A*;*B*)=0, there is no shared information between partitions *A* and *B*—they each have identified very different community structures; while if NMI(*A*;*B*)=1, the information given by partitions *A* and *B* is identical—the same community structure has been found by *A* and *B*.

### 2.4 Data

#### 2.4.1 Synthetic networks

An ensemble of 800 synthetic networks were generated to evaluate the algorithms. Networks, all with 30 row nodes and 50 column nodes, were assigned either 2 or 10 modules which are randomly positioned such that sizes of modules differed between the networks. Edge weights were then assigned to all cells within a module using random numbers derived from a skewed negative binomial distribution (following work in QuanBiMo [10]) with the dispersion parameter set to either *size*=0.5 (a network with lower connectance) or *size*=2.5 (a network with higher connectance) in both cases using a mean of 4 (see electronic supplementary material, figure S2, for histograms of these distributions). This provided four different treatments of levels of modules and connectance. Ten initial networks were calculated for each of the four treatments. Each of these 40 ‘perfectly modular’ networks was then subjected to noise introduced by rewiring a proportion of the edges in a network such that node connections are altered; in this case, the higher the level of noise, the less modular (and more random) the network structure becomes. Five replicates for four different levels of noise (noise=0,0.01,0.25,0.5) were applied to each of the 40 initial networks.

#### 2.4.2 Plant–pollinator networks

I used the 23 plant–pollinator networks [4,19–35] available in the bipartite R package (22 of which were used in [10] and the additional junker2013 network) taken from the NCEAS dataset (https://www.nceas.ucsb.edu/interactionweb/resources.html). These networks show the number of observed visitations by each recorded pollinator species to each recorded plant species at different field sites across the world. Some network properties are shown in electronic supplementary material, table S1.

### 2.5 Computing modularity

#### 2.5.1 Synthetic networks

LPAwb+ and DIRTLPAwb+ were each run once on each of the 800 quantitative synthetic networks. The performance of these algorithms at detecting weighted modular structure was assessed using three indicators: the ratio of the number of modules between the detected and synthetic networks, the ratio of modularity between the detected and synthetic networks and the NMI between that detected and that in the synthetic networks.

#### 2.5.2 Plant–pollinator networks

I computed the binary and quantitative networks for each of the datasets, removing rows and columns that contained no interaction data from the analysis. QuanBiMo, LPAwb+ and DIRTLPAwb+ were run 100 times for each binary and each weighted network in order to assess the modular structures found and the fidelity of the algorithms. I then quantified the differences between the modular structures found by the binary and weighted algorithms using the NMI criterion and investigated the differences in normalized and realized modularity.

Code implementations for the LPAwb+ and DIRTLPAwb+ algorithms are currently available online for the Julia, Matlab/Octave and R programming languages. This and the R code used to create the figures and perform the analysis presented in this paper are available in a supporting online depository [36]. For fair comparison in timing the algorithms, all computations were performed in R v. 3.2.2 using v. 2.05 of the bipartite package on an Intel(R) Core(TM) i7-5960X CPU @ 3.00 GHz desktop computer.

## 3. Results

### 3.1 Evaluating LPAwb+ and DIRTLPAwb+ on weighted networks

**Figure 2.**
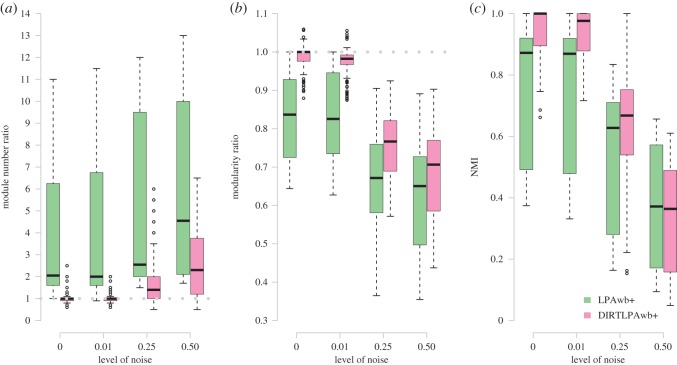
Evaluation of the LPAwb+ and DIRTLPAwb+ algorithms against synthetically generated weighted networks with known modular structure for given levels of noise. Panel (*a*) shows the ratio of detected modules to known modules, and (*b*) shows the ratio of detected modularity (*Q*_W_) to the modularity of the implanted structure. The dotted lines represent the ability to perfectly detect the synthetic community partitions. Finally, (*c*) shows the NMI between detected community structure and the embedded community structure.

Three indicators were used to assess the ability of the LPAwb+ and DIRTLPAwb+ algorithms to detect modularity in the synthetic ensemble of weighted networks, shown in figure 2. As the amount of noise in the synthetic networks increased, the ability to discern the embedded community structures decreased. However, overall DIRTLPAwb+ outperformed LPAwb+ as it was less likely to over-report the number of modules detected and more likely to identify community structure and modularity scores closer to that of those in the synthetic networks.

Community detection is affected by the level of network noise, as well as by other factors such as the number of modules and network connectance (see electronic supplementary material, figure S3). Over-reporting bias of the number of modules was reduced when the number of synthetic modules was greater, and more similar modularity scores were achieved when connectance was lower.

### 3.2 Comparing weighted modularity algorithms using plant–pollinator networks

**Figure 3.**
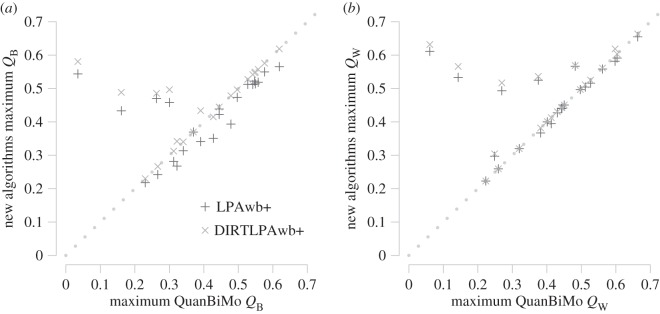
Comparing the maximum detected modularity scores by each algorithm (from 100 repetitions on each of the 23 plant–pollinator networks). The dotted line indicates a consensus, i.e. QuanBiMo and the other algorithms are in perfect correspondence. Points below the dotted line indicate QuanBiMo maximizes modularity more effectively; while points above the dotted line show that LPAwb+ or DIRTLPAwb+ detected partitions with greater modularity than QuanBiMo. Panel (*a*) shows a comparison of binary modularity scores, *Q*_B_, while (*b*) shows the weighted modularity scores, *Q*_W_.

Having shown LPAwb+ and DIRTLPAwb+ have some capacity for detecting weighted modularity, I now focus on a dataset of plant–pollinator ecological networks where these two algorithms are compared to QuanBiMo. Figure 3 shows the maximum modularity scores detected by each algorithm (from 100 replicates) for each of the networks. Full details are shown in table 1 for binary networks and table 2 for weighted networks. As expected (by definition), DIRTLPAwb+ scores were always equal to or greater than those detected by LPAwb+. Each algorithm detected similar maximum modularity scores for each network, with the exception of the datasets of kato1990, junker2013, barrett1987 and elberling 1999 in binary networks (figure 3*a*) and kato1990, junker2013, elberling1999, kevan1970 and barrett1987 for weighted networks (figure 3*b*) in which LPAwb+ and DIRTLPAwb+ detected much greater modularity scores than QuanBiMo.

**Table 1.** Comparison of QuanBiMo, LPAwb+ and DIRTLPAwb+ algorithms on binary ecological interaction networks. *Q*_B_ is the greatest value of binary modularity from 100 replicates on the network, *M* is the corresponding number of modules found in this partition and *t* is the mean time taken (seconds) to compute each algorithm once. Numbers have been rounded to 3 d.p. Numbers shown in bold are those with the highest *Q*_B_ score.

	QuanBiMo			LPAwb+			DIRTLPAwb+		
network	*Q*_B_	*M*	*t*	*Q*_B_	*M*	*t*	*Q*_B_	*M*	*t*
Safariland	**0.558**	6	1.044	0.519	9	0.012	**0.558**	6	0.521
barrett1987	0.263	4	11.587	0.470	11	0.061	**0.486**	8	2.708
bezerra2009	**0.230**	3	1.491	0.218	5	0.011	**0.230**	3	0.186
elberling1999	0.300	5	26.208	0.458	22	0.187	**0.497**	14	17.22
inouye1988	**0.427**	9	17.277	0.351	31	0.337	0.415	6	40.224
junker2013	0.161	7	42.724	0.433	55	3.06	**0.489**	19	328.953
kato1990	0.035	5	1551.827	0.544	74	13.243	**0.581**	32	5510.003
kevan1970	0.391	6	29.179	0.341	23	0.228	**0.434**	5	20.781
memmott1999	0.322	4	10.677	0.268	19	0.145	**0.342**	5	11.714
mosquin1967	**0.479**	6	0.992	0.393	11	0.015	**0.479**	6	0.774
motten1982	**0.313**	6	2.394	0.281	10	0.028	**0.313**	6	1.284
olesen2002aigrettes	**0.340**	4	1.063	0.314	7	0.010	**0.340**	4	0.303
olesen2002flores	**0.444**	4	0.923	0.422	7	0.008	**0.444**	4	0.216
ollerton2003	**0.445**	6	7.282	0.439	8	0.038	**0.445**	6	1.383
schemske1978	**0.370**	6	1.778	**0.370**	6	0.009	**0.370**	6	0.197
small1976	**0.266**	5	1.684	0.242	8	0.019	**0.266**	5	0.729
vazarr	**0.542**	7	1.348	0.512	9	0.014	**0.542**	7	0.576
vazcer	**0.619**	6	1.970	0.565	9	0.014	**0.619**	6	0.586
vazllao	**0.576**	6	1.103	0.550	8	0.015	**0.576**	6	0.542
vazmasc	**0.547**	6	1.27	0.522	8	0.010	**0.547**	6	0.342
vazmasnc	**0.527**	6	2.184	0.512	8	0.015	**0.527**	6	0.508
vazquec	**0.497**	4	1.412	0.474	7	0.011	**0.497**	4	0.311
vazquenc	**0.549**	5	0.855	0.514	7	0.009	**0.549**	5	0.254

**Table 2.** Comparison of QuanBiMo, LPAwb+ and DIRTLPAwb+ algorithms on weighted ecological interaction networks. *Q*_W_ is the greatest value of weighted modularity from 100 replicates on the network, *M* is the corresponding number of modules found in this partition and *t* is the mean time taken (seconds) to compute each algorithm once. Numbers have been rounded to 3 d.p. Numbers shown in bold are those with the highest *Q*_W_ score.

	QuanBiMo			LPAwb+			DIRTLPAwb+		
network	*Q*_W_	*M*	*t*	*Q*_W_	*M*	*t*	*Q*_W_	*M*	*t*
Safariland	**0.430**	5	1.193	0.427	7	0.012	**0.430**	5	0.347
barrett1987	0.482	4	8.898	0.567	9	0.049	**0.569**	7	1.889
bezerra2009	**0.223**	5	1.69	**0.223**	5	0.012	**0.223**	5	0.19
elberling1999	0.270	6	25.367	0.493	18	0.129	**0.517**	12	10.362
inouye1988	0.598	9	22.543	0.582	22	0.186	**0.619**	14	15.785
junker2013	0.143	6	91.534	0.533	33	1.335	**0.566**	16	102.635
kato1990	0.061	5	2210.35	0.611	48	6.217	**0.632**	20	1000.365
kevan1970	0.375	3	33.674	0.525	10	0.094	**0.536**	5	4.525
memmott1999	0.249	5	13.261	0.297	10	0.075	**0.305**	6	3.420
mosquin1967	**0.444**	6	0.935	0.440	7	0.009	**0.444**	6	0.252
motten1982	**0.382**	4	3.038	0.367	6	0.019	**0.382**	4	0.434
olesen2002aigrettes	**0.259**	5	1.083	**0.259**	5	0.007	**0.259**	5	0.117
olesen2002flores	**0.497**	5	0.938	**0.497**	5	0.006	**0.497**	5	0.085
ollerton2003	**0.413**	6	5.879	0.395	7	0.021	**0.413**	6	0.587
schemske1978	**0.320**	4	2.26	**0.320**	4	0.012	**0.320**	4	0.011
small1976	**0.527**	8	1.861	0.516	11	0.024	0.525	7	1.057
vazarr	**0.442**	6	1.672	0.441	7	0.013	**0.442**	6	0.359
vazcer	**0.604**	6	2.184	0.591	7	0.014	**0.604**	6	0.389
vazllao	**0.561**	6	1.335	0.558	8	0.012	**0.561**	6	0.418
vazmasc	**0.663**	6	1.424	0.655	7	0.009	**0.663**	6	0.301
vazmasnc	**0.401**	6	2.162	0.400	7	0.012	**0.401**	6	0.335
vazquec	**0.511**	6	2.027	0.504	7	0.013	**0.511**	6	0.350
vazquenc	**0.450**	4	0.828	**0.450**	4	0.007	**0.450**	4	0.006

Table 1 shows the greatest modularity scores detected by each algorithm, the number of modules in these partitions and the average execution time for each algorithm in the analysis of binary networks. The same partition was found by all three algorithms in only the schemske1978 network; both QuanBiMo and DIRTLPAwb+ found the same partitions for another 16 networks; while DIRTLPAwb+ found the greatest modularity score for six networks and QuanBiMo found the best modularity score in the inouye1988 network. LPAwb+ was by far the algorithm with the quickest execution time. DIRTLPAwb+ performs faster on small networks than QuanBiMo and more slowly on larger networks; however it generally found a much greater modularity score than QuanBiMo for these networks. The partitions found by LPAwb+ had more modules than those found by the solution with the greatest modularity.

For weighted networks, table 2 shows there were five networks for which the same maximum modularity was detected by all three algorithms, 10 networks in which QuanBiMo and DIRTLPAwb+ found the greatest modularity, seven networks for which DIRTLPAwb+ found the greatest modularity and a single network, small1976, that was maximized by QuanBiMo. QuanBiMo had a similar average performance time to the binary networks, with LPAwb+ finding modularity more quickly in weighted than in binary networks. DIRTLPAwb+ has a similar performance time for smaller networks as under binary conditions and performs faster for the larger networks—which can be ascribed to the lower number of modules detected by LPAwb+ for the weighted networks. LPAwb+ detects partitions which generally have more modules than that with the greatest modularity, while QuanBiMo generally finds partitions with fewer modules than the solution found with greatest modularity.

**Figure 4.**
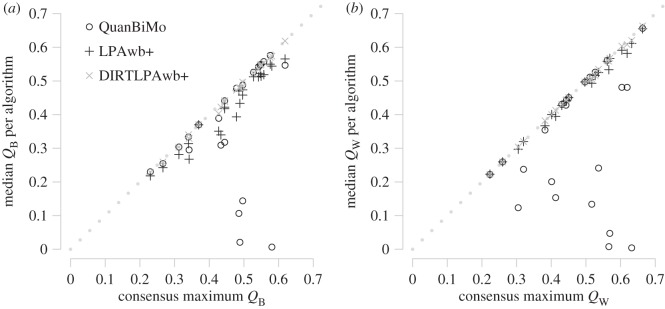
Comparison of median modularity scores found by each algorithm (from 100 repetitions on each of the 23 plant–pollinator networks) to the maximum of the modularity scores found across the algorithms—the consensus maximum modularity. Panel (*a*) shows results for binary networks, while (*b*) shows the results for weighted networks. The dotted line represents algorithm efficacy, where median modularity score is equal to the maximum consensus modularity score that was detected.

Figure 4 shows the median detected modularity scores for each algorithm against the overall maximum modularity score for each network. Figure 4*a* shows that DIRTLPAwb+ consistently finds modularity scores closest to the maximal value, that LPAwb+ scores were close, but not so close and that while QuanBiMo could achieve consistency as good as the DIRTLPAwb+, for several networks QuanBiMo had a median value much lower than the maximum modularity detected. Similarly in figure 4*b*, DIRTLPAwb+ shows high consistency as does LPAwb+ (more so than for binary networks), while QuanBiMo in general performs less consistently for weighted networks than binary networks.

**Figure 5.**
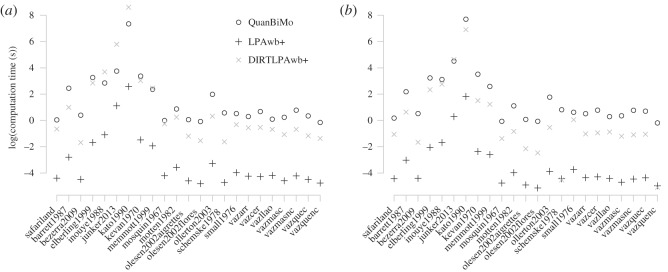
Average computational time for each algorithm (measured over 100 replicates) on the (*a*) binary and (*b*) quantitative representations of each plant–pollinator network.

The average time to run each algorithm is shown in figure 5. Performance time is network dependent; where it takes longer to compute and report modularity for larger networks. LPAwb+ performed the quickest on all networks by roughly 2 orders of magnitude. Performance on the binary (figure 5*a*) and quantitative (figure 5*b*) network representations was similar. However, QuanBiMo performed faster for binary (rather than quantitative) inputs on 17 of the 23 networks. On the other hand, LPAwb+ ran quicker with quantitative network representations (19 out of 23), as did DIRTLPAwb+ (20 out of 23). For the five cases where DIRTLPAwb+ took longer than QuanBiMo, DIRTLPAwb+ found a partition with greater modularity four times and QuanBiMo found the greatest modularity score once (the binary representation of inouye1988).

### 3.3 Differences in community structure between the algorithms

For each algorithm, the community partitions achieving the greatest modularity scores on each network were compiled and then compared against those found by the other algorithms using NMI. The results of these pairwise comparisons are shown in table 3. Cells are highlighted when one algorithm detected modularity scores greater than the opposing algorithm (see tables 1 and 2) and NMI is given as a range when one of the algorithms detected multiple partitions resulting in its largest modularity score (see column U in electronic supplementary material, tables S2 and S3). In almost all cases where the same greatest modularity score was detected, this corresponded to the same community partition. The exception is the binary vazmasc network where two solutions were identified by both QuanBiMo and DIRTLPAwb+. These solutions are similar as the given NMI scores are high.

**Table 3.** Pairwise comparison of shared information between the detected community partitions with the greatest modularity score for each algorithm. For both binary and quantitative cases, the configurations that found the greatest modularity scores were found for each algorithm and compared using NMI. NMI is 1 when the modular configurations are equivalent, while a score of 0 indicates no shared information. Cells are highlighted to show which of the two algorithms in each comparison found partitions resulting in the greatest modularity score (QuanBiMo in blue, DIRTLPAwb+ in pink and LPAwb+ in green) as shown in tables 1 and 2. Where no highlighting is applied, both algorithms detected community partitions resulting in the same modularity score. When the value of a cell is given as a range, at least one of the competing algorithms found multiple network configurations resulting in its maximum modularity (see electronic supplementary material, tables S2 and S3) and so the minimum and maximum NMI scores across all such configurations between the algorithms are shown.

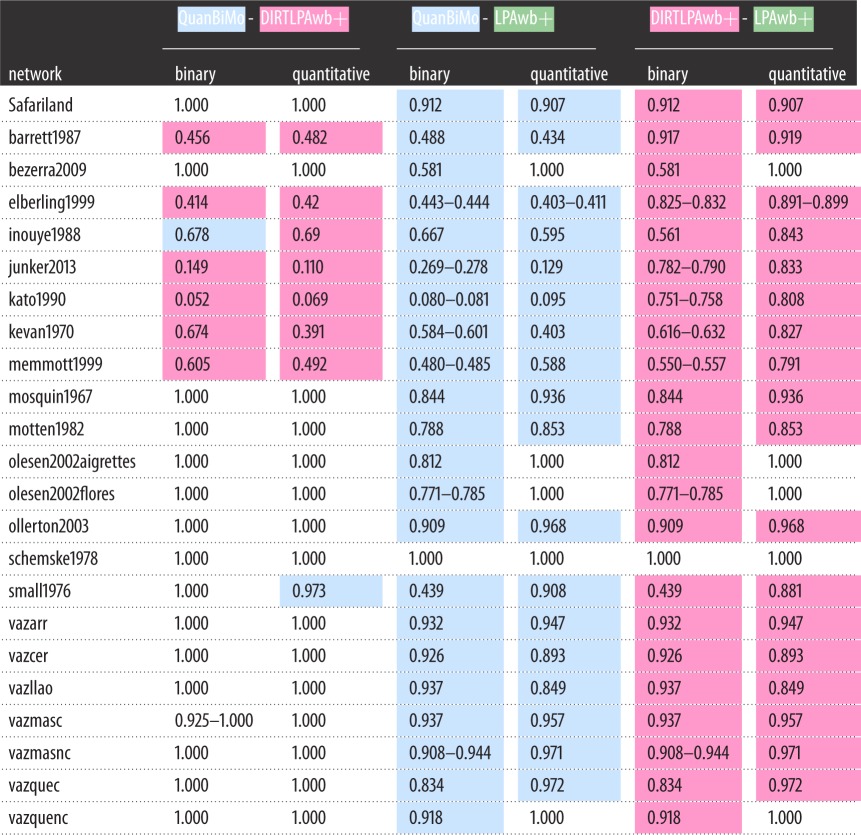

Where the differences in modularity scores detected by QuanBiMo and DIRTLPAwb+ were greatest also corresponded to greater differences in the community partitions being identified. In general, the community partitions identified by the LPAwb+ algorithm were found to be more similar to those found by DIRTLPAwb+ than QuanBiMo, which is perhaps unsurprising given the similarities in the algorithms themselves.

Details of the actual partitions for the plant–pollinator networks evaluated in table 3 are provided in the electronic supplementary material.

### 3.4 Contrasting binary and quantitative modular structure

**Figure 6.**
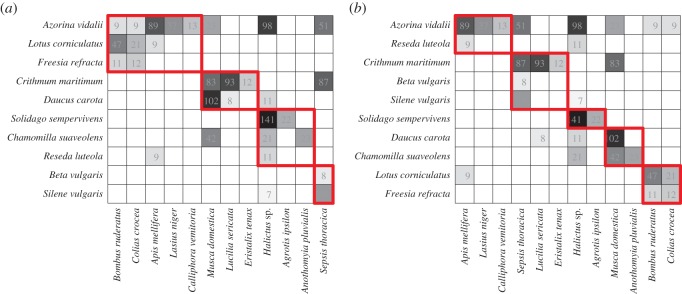
A visual comparison of the modular structures identified for the olesen2002flores dataset of plant–pollinator visitations as a (*a*) binary (*Q*_B_=0.444, four modules, *Q*^norm^_B_=0.625) and (*b*) quantitative (*Q*_W_=0.497 , five modules, $Q_{W}^{\mathrm{norm}}=0.625$) network. Modulesare identified in red. The NMI shared between these two modular compositions is NMI=0.619 indicating a qualitative difference in the revealed modular structure.

Maximizing binary modularity and maximizing weighted modularity results in different identified modular structures. Figure 6*a* shows the partition with the greatest binary modularity for the olesen2002flores network, while figure 6*b* shows the partition with the greatest weighted modularity. The same dataset has qualitatively different structure between its weighted and binary representations. The shared NMI for these two partitions is NMI=0.619 indicating the identified partitions may share some similarities, but are overall quite different from each other.

**Figure 7.**
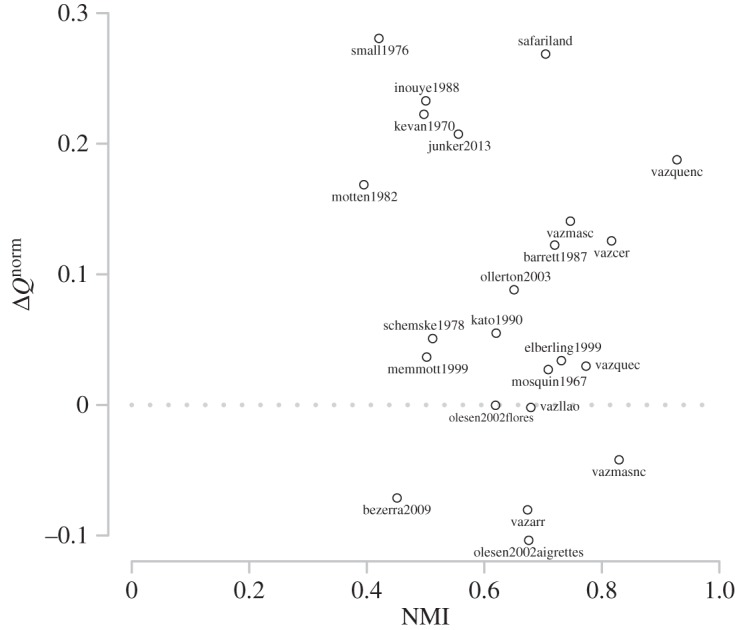
The change in normalized modularity scores found between the weighted and binary networks ($\Delta Q^{\mathrm{norm}}=Q_{W}^{\mathrm{norm}}-Q_{B}^{\mathrm{norm}}$) against the NMI between the weighted and binary partitions for each network.

Figure 7 shows the differences in normalized modularity and NMI between the binary and weighted network representations. Only three of the networks (vazquenc, vazmasnc and vazcer) have a NMI greater than 0.8—indicating major differences in identified binary and quantitative modular structures. The strength of assortative mixing, measured by normalized modularity, was generally greater in weighted than binary networks. However, four networks (olesen2002aigrettes, vazarr, bezerra2009 and vazmasnc) showed greater assortative mixing in their binary representations and for two networks (olesen2002flores and vazllao) the assortative mixing strength was nearly the same in both binary and weighted networks—though the community partitions are very different.

Not only were the detected modularity scores different between the binary and weighted networks—but the number of modules found in each partition of these networks also differed. Only eight of the networks had the same number of modules under binary and weighted conditions; while eight had more modules in the weighted networks and seven had more modules in the binary network representation (tables 1 and 2).

**Figure 8.**
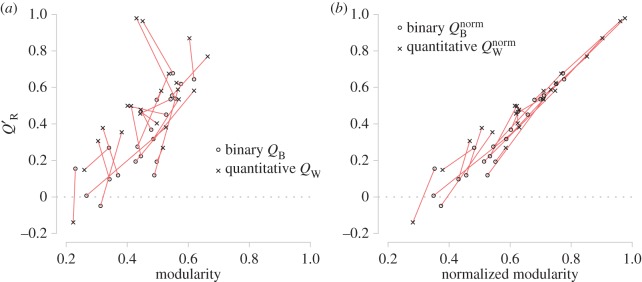
(*a*) The greatest modularity scores (*Q*_B_ and *Q*_W_) for each network and their corresponding realized modularity scores (*Q*′_R_). (*b*) The normalized modularity scores (*Q*^norm^_B_ and *Q*^norm^_W_) calculated using the partitions with greatest modularity scores plotted against their corresponding realized modularity scores. Each red line joins together the binary and quantitative scores of the same network.

There appears to be a weak positive relationship between realized modularity and modularity (figure 8*a*); however normalized and realized modularity appear to be much more strongly correlated (figure 8*b*). There does not appear to be a relationship between the binary and quantitative measures for each network.

## 4. Discussion

I tested the efficacy of three algorithms maximizing Dormann and Strauss's weighted version of Barber's bipartite modularity in plant–pollinator networks. One of the major results from this paper is that different modular structures were found for each of the binary and weighted representations of plant–pollinator networks. In binary networks, modules are formed by attempting to maximize the density of edges; while in quantitative networks, modules are formed that maximize the density of edge weights. In the former, strongly interacting nodes are just as important as nodes that only rarely interact; whilst in the latter, modules are likely to form around the strongest node–node interactions. It is unclear to me how to interpret these network structural differences, but I hope that presenting these data will facilitate discussion to understand what these differences mean. Binary and weighted plant–pollinator modular structures for example may both provide useful information that can be used to inform conservation policy.

It was also possible to compare and evaluate the performance of each of the three algorithms. LPAwb+ and DIRTLPAwb+ gave more consistent modularity scores than those found by QuanBiMo across the test networks. The robustness of modularity maximization algorithms is important when considering the reproducibility of results and how many times a community detection algorithm should be applied to be able to report a representative value for the maximum modularity of a network. QuanBiMo struggled to report ‘good’ modularity scores in the larger datasets. All three algorithms were able to detect greater modularity than previously reported [10], fig. 6 and generally performed well on the binary and quantitative test networks (a binary network can be seen as a special case of a quantitative network). But, QuanBiMo has the potential to fall into below par solutions and there is no diagnostic to show when this occurs. There is no guarantee that the greatest possible modularity was found in any of the test networks here; indeed, maximizing bipartite modularity is an NP-hard problem [37] and it may be difficult to find an algorithm which performs well on this problem for any possible network. While LPAwb+ was not able to maximize modularity so well as DIRTLPAwb+ or QuanBiMo on the majority of datasets (though the modularity found was near the maximal value found here), its fast performance makes it an ideal algorithm for exploratory research and for investigating modularity in larger networks, where parallelization of the algorithm [13] may become useful.

The QuanBiMo algorithm takes two input values: the number of algorithmic steps that should be performed to attempt to find greater modularity than the current partitions modularity; and the tolerance threshold for greater modularity scores. Clearly, the default values were not appropriate for some of the networks assessed here; where much greater modularity was detected by the new algorithms. However, there is no diagnostic to tell that QuanBiMo has returned a sub-par modularity value without comparisons (which may be a lengthy process); or what suitable input parameters may be for a particular network. There is a strong trade-off between computational effort and the accuracy of the returned modularity. On the other hand, LPAwb+ takes no input parameters and was able to quickly find modularity scores near to the consensus maximum modularity. DIRTLPAwb+ has two input parameters: the minimum number of modules to search for and the number of times that LPAwb+ should be initialized for each module number. By running the LPAwb+ algorithm multiple times with different initial module labels, DIRTLPAwb+ is able to explore more of the modularity landscape than LPAwb+, which allowed it to find partitions with greater modularity. Unlike LPAwb+ and QuanBiMo, DIRTLPAwb+ is allowed multiple attempts to climb the modularity landscape, from many different initial points within the modularity landscape. Additionally and unlike QuanBiMo, the parameters used by DIRTLPAwb+ have physical meaning in the context of the network—and the time complexity of this algorithm can be estimated from the number of calls that will be made to the LPAwb+ algorithm (as LPAb+'s time complexity is known [13]).

There are four challenges to address when attempting to maximize modularity [38] which are also relevant to weighted modularity. Any modularity maximization algorithm only uses information within the incidence matrix and is thus agnostic to hierarchies within the dataset—the algorithm will find communities at the resolution that has the greatest modularity it can compute; which may be different from the resolution which corresponds best with any additional information known about the network. This is further complicated as several hierarchical levels may exist within an individual network. Some work has started to address this problem in terms of visualizing the network as a multiscale structure [10,15,39], but this requires finding a suitable starting resolution. As found with QuanBiMo, the ability of algorithms to maximize modularity can be highly dependent on network properties such as size. Finally, it is recognized that the modularity landscape is ‘glassy’—there are many local modularity maxima; but detecting the global peak is extremely difficult and finding an algorithm that can capably traverse this ‘glassy’ landscape is a challenge.

A further challenge will be to find appropriate null models to test weighted modularity against in order to standardize the effect size of modularity in different networks [10]. In principle, it would be good to test against a null ensemble in which both the allowed interactions and the strength of these interactions are allowed to vary. However, in this paper, I have only focused on the optimization of weighted modularity.

Another limitation of the weighted modularity definition explored here is that it is only valid on networks where all connections are positive. However, methods have been created to search for modules in weighted networks with positive and negative link strengths in unipartite networks that could easily be extended for bipartite networks [40].

I focused on a specific definition of modularity in this paper—but note that others do exist [8,41]. Thébault [9] compared two binary bipartite modularity based measures that have been applied in ecology and concluded that different forms of modularity may be useful in different contexts; but that the form of modularity used here [7,10] corresponded well with that for unipartite networks [6,11]—and is well suited for identifying densely connected modules. Other modularity measures [8,41] do not identify joint communities made of both types of nodes—but rather identify communities within each type of node, though neither of these approaches has yet been extended to weighted networks to my knowledge.

The major advantage in a definition of weighted modularity is that it allows for much more information about a network to be used to detect communities. Both binary and weighted measurements contain different information about a network and may be useful—though I expect weighted measurements may in general contain more relevance for the analysis of real world networks—the strength of interactions is undoubtedly an important component of network structure. Other modularity definitions and their weighted extensions are also in need of further investigation to consider communities within each type of node and how these may overlap with the joint communities considered here.

Additionally, I looked at two alternative ways of reporting modularity: normalized modularity and realized modularity. Normalized modularity measures the strength of assortative mixing and is a useful network index that can be used as a comparison indicator across different network studies. Modularity by itself is often used as a network indicator—but this is not appropriate when comparing different networks whose theoretical modularity maxima may differ. I find normalized modularity is strongly correlated with the proportion of within module interactions (realized modularity) which is an intuitive way for understanding modularity. I recommend future studies investigating modularity to report one or both of these measures.

## 5. Conclusion

Real-world networks are not formed of binary interactions. I encourage researchers to apply weighted modularity measures to their datasets and evaluate the community partitions that are identified.

LPAwb+ is an algorithm that would be well suited for exploratory analysis and use on large networks—as it is fast and, while it did not return the best modularity values of the methods tested here, the solutions it did find were consistently high. Care has to be taken with both QuanBiMo and DIRTLPAwb+ in setting appropriate input parameter settings such that the analysis is not computationally infeasible. I would recommend using DIRTLPAwb+ over QuanBiMo, as DIRTLPAwb+ has more meaningful input parameters, can perform no worse than LPAwb+ and its performance was less variable than QuanBiMo on the networks tested in this study.

I have made the code for the LPAwb+ and DIRTLPAwb+ algorithms as well as the analysis performed in this paper available online [36] to allow researchers to replicate my findings and encourage those with access to potentially interesting weighted bipartite datasets to analyse them using these methods.

## Supplementary Material

Supporting Information: Improved community detection in weighted bipartite networks

## Data accessibility

Codes used in this paper are available on Github: https://github.com/sjbeckett/weighted-modularity-LPAwbPLUS and are archived on Zenodo [36].
