# Supplementary material for: Improved community detection in weighted bipartite networks
Source: R Soc Open Sci. 2016 Jan 20;3(1):140536. doi: 10.1098/rsos.140536 (PMC4736915; doi:10.1098/rsos.140536)
Supplement: Supporting Information: Improved community detection in weighted bipartite networks [file rsos140536supp1.pdf]

Supporting Information: Improved community detection in weighted bipartite networks

Stephen Beckett

Biosciences, College of Life and Environmental Sciences, University of Exeter, EX4 4QE

Current address: School of Biology, Georgia Institute of Technology, Atlanta, Georgia, USA

Contents

1 Synthetic Networks 2

2 Plant-pollinator datasets 4

3 Details for viewing plant-pollinator partitions 6

4 Extra results from algorithm analysis on plant-pollinator datasets 7

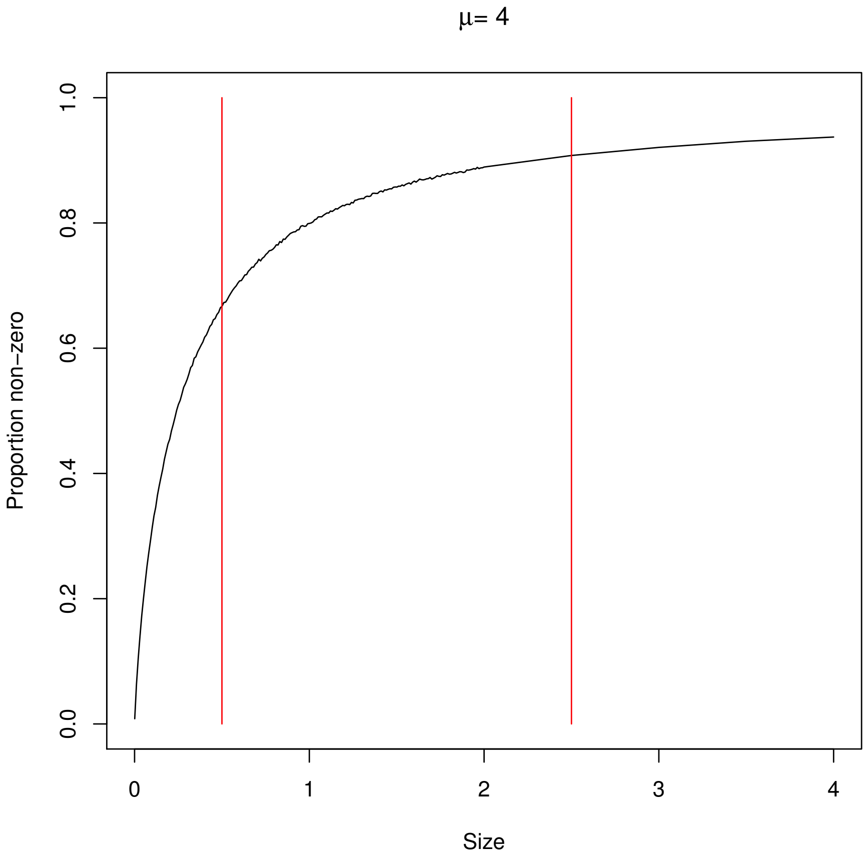

Figure 1: Proportion of 10,000 numbers drawn from negative binomial distributions with a mean of 4 that were not equal to zero.

1 Synthetic Networks

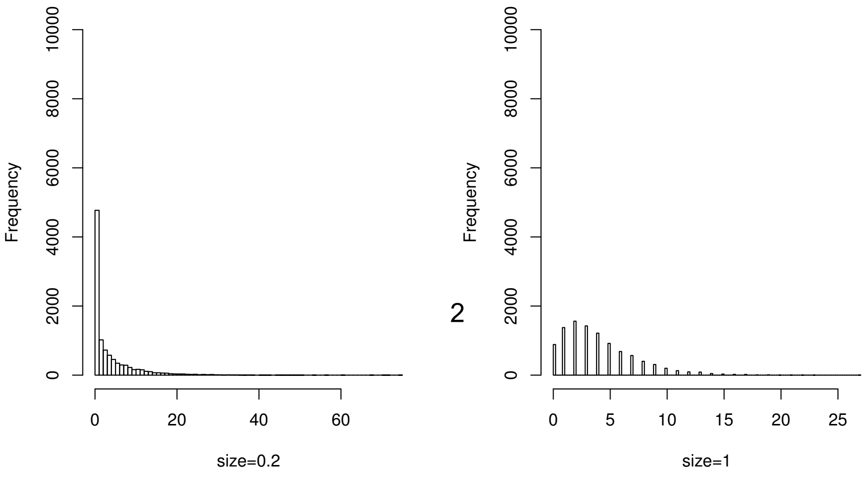

Figure 2: Histograms of 10,000 draws from the two negative binomial distribu-

The results from the synthetic ensemble are saved in the folder paper/paper-code/syntheticEnsemble.RData in the supporting data repository [1].

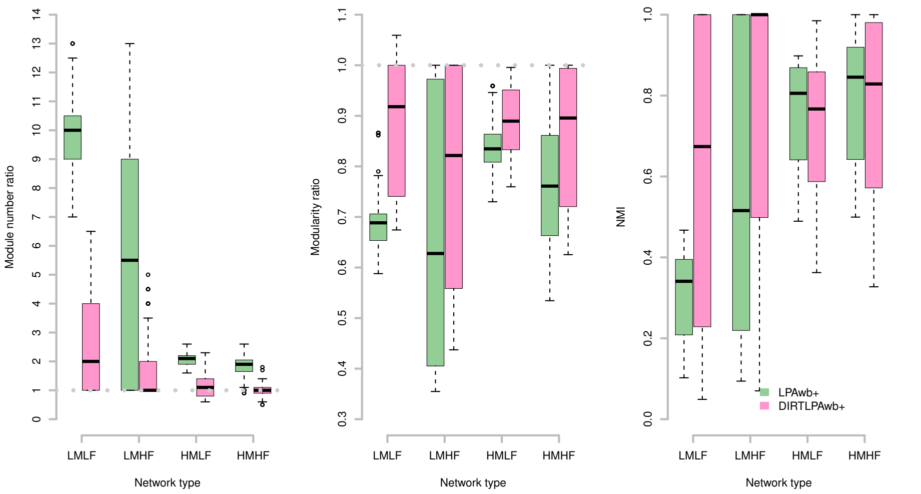

Figure 3: Evaluation of the LPAwb+ and DIRTLPAwb+ algorithms against synthetically generated weighted networks with known modular structure for four different treatments: LMLF – 2 modules and low connectance (dispersion parameter given as $size = 0.5$), LMHF – 2 modules, higher connectance(dispersion parameter given as $size = 2.5$), HMLF – 10 modules, lower connectance, HMHF – 10 modules, higher connectance . (a) shows the ratio of detected modules to known modules , (b) shows the ratio of detected modularity (Q_W) to the modularity of the implanted structure. The dotted lines represent the ability to perfectly detect the synthetic community partitions. Finally (c) shows the normalised mutual information (NMI) between detected community structure and the embedded community structure.

2 Plant-pollinator datasets

Network	Rows	Columns	Edges	Fill	Reference
Safariland	9	27	39	1130	[2, 3, 4]
barrett1987	12	102	167	550	[5]
bezerra2009	13	13	71	28224	[6]
elberling1999	23	118	238	383	[7]
inouye1988	41	83	268	1459	[8]
junker2013	56	257	572	3053	[9]
kato1990	91	679	1206	2392	[10]
kevan1970	30	114	312	2523	[11]
memmott1999	25	79	299	2183	[12]
mosquin1967	11	18	38	134	[13]
motten1982	13	44	143	2225	[14]
olesen2002aigrettes	14	13	52	1512	[15]
olesen2002flores	10	12	30	1139	[15]
ollerton2003	9	56	103	594	[16]
schemske1978	7	32	59	299	[17]
small1976	13	34	141	992	[18]
vazarr	10	29	43	515	[2, 3, 4]
vazcer	9	33	45	613	[2, 3, 4]
vazllao	10	29	42	677	[2, 3, 4]
vazmasc	8	26	36	286	[2, 3, 4]
vazmasnc	8	35	51	719	[2, 3, 4]
vazquec	8	27	47	592	[2, 3, 4]
vazquenc	7	24	31	761	[2, 3, 4]

Table 1: Network properties of the datasets used in this study.

3 Details for viewing plant-pollinator partitions

The modular partitions found for each plant-pollinator network for both binary and quantitative cases are described in the supporting data repository [1].

They can be found by navigating to paper/papercode/output/configurations

Each file in this folder is of a given format:

First the network name is given

Second a letter B or Q details whether this is a binary or quantitative network partition

Next are two sets of two letters, which together describe the two algorithms being compared (LP: LPAwb+, EX: DIRTLPAwb+, QB: QuanBiMo)

Then the final string of important characters again identifies whether it is binary or quantitative and the corresponding algorithm the partition was found by. If this string is preceded by either min or max - this means that multiple network configurations were found with the highest modularity score detected by one of the two competing algorithms (see column U in tables S2-S3). min and max correspond to partitions that either minimised or maximised the NMI score between the solutions each algorithm identified. If neither min or max are listed there is no range of NMI values – as identified in Table 3 in the main text.

Within each file the list of all row and column nodes is given at depth 0, whilst at depth 1 the modular configurations the nodes are assigned to is listed.

4 Extra results from algorithm analysis on plant-pollinator datasets

Network	QuanBiMo					LPAwb+					Exhaustive LPAwb+				
	R	$\tilde{x}$	U	F	$Q_R^{'}$	R	$\tilde{x}$	U	F	$Q_R^{'}$	R	$\tilde{x}$	U	F	$Q_R^{'}$
Safariland	89	0.558	1	0	0.538	100	0.519	2	0	0.385	46	0.554	1	0	0.538
barrett1987	1	0.106	1	0	0.521	95	0.470	1	0	0.246	4	0.480	1	0	0.317
bezerra2009	76	0.230	1	0	0.155	100	0.218	1	0	-0.099	34	0.229	1	0	0.155
elberling1999	1	0.144	1	6	0.395	100	0.458	2	0	0.008	1	0.483	1	0	0.193
inouye1988	1	0.389	1	0	0.194	55	0.351	1	0	-0.179	1	0.403	1	0	0.209
junker2013	1	0.021	1	0	0.570	62	0.433	33	0	-0.091	1	0.480	1	0	0.119
kato1990	1	0.006	1	0	0.945	98	0.544	90	0	0.154	1	0.573	1	0	-0.987
kevan1970	1	0.309	1	1	0.301	8	0.341	3	0	-0.205	1	0.423	1	0	0.276
memmott1999	1	0.295	1	0	0.171	60	0.268	8	0	-0.344	1	0.329	1	0	0.097
mosquin1967	58	0.479	1	0	0.368	100	0.393	1	0	-0.053	42	0.472	1	0	0.368
motten1982	3	0.304	1	0	-0.049	100	0.281	1	0	-0.259	3	0.303	1	0	-0.049
olesen2002aigrettes	21	0.334	1	0	0.269	95	0.314	1	0	0.000	75	0.340	1	0	0.269
olesen2002flores	25	0.441	1	0	0.467	95	0.422	2	0	0.133	44	0.441	1	0	0.467
ollerton2003	5	0.318	1	2	0.223	39	0.439	1	0	0.165	5	0.439	1	0	0.223
schemske1978	54	0.370	1	0	0.119	100	0.370	1	0	0.119	100	0.370	1	0	0.119
small1976	5	0.255	1	0	0.007	99	0.242	1	0	-0.234	8	0.260	1	0	0.007
vazarr	100	0.542	1	0	0.535	100	0.512	1	0	0.395	13	0.535	1	0	0.535
vazcer	29	0.547	1	0	0.644	100	0.565	1	0	0.467	75	0.619	1	0	0.644
vazllao	100	0.576	1	0	0.619	78	0.550	2	0	0.524	27	0.570	1	0	0.619
vazmasc	100	0.547	2	0	0.556	100	0.522	1	0	0.444	56	0.547	2	0	0.556
vazmasnc	10	0.526	1	0	0.451	100	0.512	2	0	0.373	20	0.522	1	0	0.451
vazquec	26	0.488	1	0	0.532	100	0.474	1	0	0.234	77	0.497	1	0	0.532
vazquenc	100	0.549	1	0	0.677	100	0.514	1	0	0.548	78	0.549	1	0	0.677

Table 2: Extra results from the evaluations of the binary version of these networks. R is the number of times that the best partitions (with highest Q_B) were found from the 100 tests, $\tilde{x}$ is the median Q_B score, U is the number of unique configurations found with the maxmium Q_B score (for each method) judged by comparing the normalised mutual information of partitions sharing this value, F is number of times that the algorithms reported a failure (from the 100 runs) and $Q_R^{'}$ is the realised modularity of the partition with highest Q_B score (for each method). Numbers have been rounded to 3 d.p.

	QuanBiMo					LPAwb+					Exhaustive LPAwb+				
Network	R	$\tilde{x}$	U	F	$Q^{'}_{R}$	R	$\tilde{x}$	U	F	$Q^{'}_{R}$	R	$\tilde{x}$	U	F	$Q^{'}_{R}$
Safariland	75	0.430	1	0	0.979	100	0.427	1	0	0.963	35	0.430	1	0	0.979
barrett1987	1	0.047	1	0	0.844	100	0.567	1	0	0.560	3	0.568	1	0	0.535
bezerra2009	22	0.222	1	0	-0.139	100	0.223	1	0	-0.139	100	0.223	1	0	-0.139
elberling1999	1	0.134	1	3	0.436	100	0.493	4	0	0.180	1	0.506	1	0	0.269
inouye1988	1	0.481	1	0	0.589	100	0.582	1	0	0.406	1	0.608	1	0	0.582
junker2013	1	0.008	1	0	0.597	100	0.533	1	0	0.452	1	0.558	1	0	0.588
kato1990	1	0.004	1	0	0.896	100	0.611	1	0	0.355	1	0.620	1	0	-0.967
kevan1970	1	0.241	1	0	0.559	100	0.525	1	0	0.583	5	0.535	1	0	0.675
memmott1999	1	0.123	1	0	0.407	100	0.297	1	0	0.132	2	0.304	1	0	0.306
mosquin1967	73	0.444	1	0	0.478	100	0.440	1	0	0.403	60	0.444	1	0	0.478
motten1982	12	0.354	1	0	0.355	100	0.367	1	0	0.212	97	0.382	1	0	0.355
olesen2002aigrettes	91	0.259	1	0	0.148	100	0.259	1	0	0.148	100	0.259	1	0	0.148
olesen2002flores	72	0.497	1	0	0.403	100	0.497	1	0	0.403	100	0.497	1	0	0.403
ollerton2003	1	0.153	1	0	0.498	100	0.395	1	0	0.431	88	0.413	1	0	0.498
schemske1978	1	0.238	1	0	0.378	100	0.320	1	0	0.378	100	0.320	1	0	0.378
small1976	35	0.526	1	0	0.381	100	0.516	1	0	0.260	1	0.516	1	0	0.387
vazarr	22	0.428	1	0	0.456	100	0.441	1	0	0.449	60	0.442	1	0	0.456
vazcer	31	0.481	1	0	0.869	100	0.591	1	0	0.830	60	0.604	1	0	0.869
vazllao	100	0.561	1	0	0.625	100	0.558	1	0	0.586	34	0.561	1	0	0.625
vazmasc	27	0.656	1	0	0.769	100	0.655	1	0	0.727	62	0.663	1	0	0.769
vazmasnc	25	0.201	1	0	0.499	100	0.400	1	0	0.497	20	0.400	1	0	0.499
vazquec	56	0.511	1	0	0.581	100	0.504	1	0	0.544	14	0.504	1	0	0.581
vazquenc	100	0.450	1	0	0.963	100	0.450	1	0	0.963	100	0.450	1	0	0.963

Table 3: Extra results from the evaluations of the weighted version of these networks. R is the number of times that the best partitions (with highest Q_W) were found from the 100 tests, $\tilde{x}$ is the median Q_W score, U is the number of unique configurations found with the maxmium Q_W score (for each method) judged by comparing the normalised mutual information of partitions sharing this value, F is number of times that the algorithms reported a failure (from the 100 runs) and $Q^{'}_{R}$ is the realised modularity of the partition with highest Q_W score (for each method). Numbers have been rounded to 3 d.p.

References

[1] S. J. Beckett, “weighted-modularity-LPAwbPLUS: Improved community detection in weighted bipartite networks,” p. 10.5281/zenodo.34055, 2015.

[2] D. P. Vázquez and D. Simberloff, “Ecological specialization and susceptibility to disturbance: conjectures and refutations,” The American Naturalist, vol. 159, no. 6, pp. 606–623, 2002.

[3] D. P. Vázquez, Interactions among introduced ungulates, plants, and pollinators: a field study in the temperate forest of the southern Andes. PhD thesis, University of Tennessee, Knoxville, Tennessee, USA., 2002.

[4] D. P. Vázquez and D. Simberloff, “Changes in interaction biodiversity induced by an introduced ungulate,” Ecology Letters, vol. 6, no. 12, pp. 1077–1083, 2003.

[5] S. C. Barrett and K. Helenurm, “The reproductive biology of boreal forest herbs. i. breeding systems and pollination,” Canadian Journal of Botany, vol. 65, no. 10, pp. 2036–2046, 1987.

[6] E. L. Bezerra, I. C. Machado, and M. A. Mello, “Pollination networks of oil-flowers: a tiny world within the smallest of all worlds,” Journal of Animal Ecology, vol. 78, no. 5, pp. 1096–1101, 2009.

[7] H. Elberling and J. M. Olesen, “The structure of a high latitude plant-flower visitor system: the dominance of flies,” Ecography, vol. 22, no. 3, pp. 314–323, 1999.

[8] D. W. Inouye and G. H. Pyke, “Pollination biology in the snowy mountains of australia: comparisons with montane colorado, usa,” Australian Journal of Ecology, vol. 13, no. 2, pp. 191–205, 1988.

[9] R. R. Junker, N. Blüthgen, T. Brehm, J. Binkenstein, J. Paulus, H. Martin Schaefer, and M. Stang, “Specialization on traits as basis for the niche-breadth of flower visitors and as structuring mechanism of ecological networks,” Functional Ecology, vol. 27, no. 2, pp. 329–341, 2013.

[10] M. Kato, T. Kakutani, T. Inoue, and T. Itino, “Insect-flower relationship in the primary beech forest of ashu, kyoto: an overview of the flowering phenology and the seasonal pattern of insect visits,” Contributions from the Biological Laboratory, Kyoto University, 1990.

[11] P. Kevan, “High arctic insect-flower visitor relations: the inter-relationships of arthropods and flowers at lake hazen, ellesmere island, northwest territories, canada,” PhD thesis, 1970.

[12] J. Memmott, “The structure of a plant-pollinator food web,” Ecology Letters, vol. 2, no. 5, pp. 276–280, 1999.

[13] T. Mosquin and J. Martin, “Observations on the pollination biology of plants on melville island, nwt, canada,” Canadian Field Naturalist, vol. 81, pp. 201–205, 1967.

[14] A. F. Motten, “Pollination ecology of the spring wildflower community of a temperate deciduous forest,” Ecological Monographs, pp. 21–42, 1986.

[15] J. M. Olesen, L. I. Eskildsen, and S. Venkatasamy, “Invasion of pollination networks on oceanic islands: importance of invader complexes and endemic super generalists,” Diversity and Distributions, vol. 8, no. 3, pp. 181–192, 2002.

[16] J. Ollerton, S. D. Johnson, L. Cranmer, and S. Kellie, “The pollination ecology of an assemblage of grassland asclepiads in south africa,” Annals of Botany, vol. 92, no. 6, pp. 807–834, 2003.

[17] D. W. Schemske, M. F. Willson, M. N. Melampy, L. J. Miller, L. Verner, K. M. Schemske, and L. B. Best, “Flowering ecology of some spring woodland herbs,” Ecology, pp. 351–366, 1978.

[18] E. Small, “Insect pollinators of the mer bleue peat bog of ottawa,” Canadian field-naturalist, 1976.
